# Public Policy Versus Individual Rights and Responsibility: An Economist’s Perspective

**Published:** 2011-08-15

**Authors:** Frank J. Chaloupka

**Affiliations:** University of Illinois

## Abstract

Interventions to reduce childhood obesity entail ethical considerations. Although a rationale exists for government to intervene in a way that limits individual rights while protecting the public's health, a clear economic rationale also exists. The markets for goods and services that contribute to obesity are characterized by multiple failures that create an economic rationale for government to intervene (eg, consumers' lack of accurate information regarding obesogenic foods and beverages). If effective public policies for reducing obesity and its consequences are to be developed and implemented, individual rights and government interests must be balanced.

## Introduction

As discussed in the diverse set of articles in this issue of *Preventing Chronic Disease*, substantial ethical considerations are entailed in interventions to reduce childhood obesity (1). Phillips et al (2) succinctly describe ethical concerns related to population-based public policy interventions to curb childhood obesity, focusing on those surrounding Arkansas' 2003 comprehensive legislation regarding school-based interventions (3). The concerns raised regarding Act 1220 included privacy matters regarding the measurement and reporting of body mass index (BMI) and the time and economic burden the act's requirements place on schools. However, as Phillips et al describe, a rationale exists for government to intervene in a way that limits individual rights while protecting the public's health.

## Economic Rationale for Action to Curb Childhood Obesity

In addition to the public health rationale that supports policy interventions to curb obesity, a clear economic rationale exists for such interventions. One of the key assumptions that underlies economic theory and analysis is that of consumer sovereignty — that is, the individual consumer knows what is best for himself or herself. This results in the conclusion that free, competitive markets are most efficient in allocating society's scarce resources and that these markets, unfettered by government regulation, will produce the best outcomes. Moreover, the assumption is that consumers are making their decisions based on full information and that they bear all costs and benefits of their consumption and production choices. The markets for goods and services that contribute to obesity, however, are characterized by multiple failures that create an economic rationale for governments to intervene (4,5).

First, consumers have imperfect information about the consequences of certain behaviors they engage in that contribute to obesity. They lack accurate or thorough information regarding the calorie and nutrient content of foods and beverages they consume, do not fully grasp the benefits of physical activity, and underappreciate the health and economic consequences of being overweight or obese. This is most likely to be true during childhood and adolescence, when diet and activity habits that carry forward into adulthood are established. These decisions are further distorted by pervasive marketing efforts that increase the perceived benefits of consumption of such unhealthful options as sugar-sweetened beverages and fast foods.

A second market failure results from what economists refer to as *time inconsistent preferences* — the idea that what a person prefers at 1 time is inconsistent with that person's preferences at a different time (6). This time inconsistency is prominent with behaviors that provide immediate gratification but have long-term health and other consequences (eg, dieters whose long-term goal is to maintain a healthy weight but who engage in binge-eating episodes that keep them from doing so, despite regretting these choices later). Again, this is particularly problematic for children and adolescents, who place a higher value on their immediate gratification while heavily discounting the long-term health consequences of their behaviors.

An additional market failure results from the externalities present in these markets — the costs that are not borne by those who produce or consume, but rather by others in society. Most notable are the financial externalities (monetary costs) that result from using public funds to treat the diseases caused by an unhealthful diet, physical inactivity, and obesity. Recent estimates put the rapidly increasing health care costs of treating obesity at $147 billion in 2006, and a considerable share of these monies were paid through Medicare and Medicaid (7). The costs are substantially higher when the lost productivity caused by obesity is included. One study estimated that the annual cost of obesity to an obese person is $8,365 for a man and $6,518 for a woman (8). To the extent that time inconsistent preferences contribute to choices that generate these costs, at least some of the costs borne by individual consumers can be treated as externalities. These externalities result in a disconnect between the costs faced by consumers and the benefits they receive and the social costs and benefits of their behaviors. For example, the price of sugar-sweetened beverages does not reflect the full costs associated with their consumption, given the shared costs of treating the obesity, diabetes, cardiovascular diseases, and other consequences that result from their consumption (9). In contrast, the benefits that a supermarket owner receives from locating in a food desert (areas characterized by poor access to healthful and affordable food) are almost certainly less than societal benefits, given that access to fresh fruits and vegetables and other more healthful options has been linked to better weight outcomes, fewer negative health consequences, and reduced health care costs (10).

In economics, the first-best policy options are those that target specific market failures. In the case of information failures, multiple interventions are available. A policy similar to the BMI measurement and reporting policy in Arkansas can increase awareness among parents of what their child's healthy weight should be, and school-based education efforts that integrate nutrition and activity into the curricula can increase children's knowledge. Policies that address information failures among the broader population include menu-labeling laws that mandate provision of calorie and other nutrition information on fast-food menu boards and full-service restaurant menus and more comprehensive nutrition labeling on packaged goods. Meanwhile, policies aimed at reducing exposure to potentially misleading information can be adopted (eg, limits on advertising of unhealthful foods and beverages, particularly to children, who are more susceptible to these messages and less able to process them; strong standards for using descriptors such as *low-fat* or *light* that can misrepresent overall nutritional quality). Similarly, bans on sugar-sweetened beverages in school vending machines or the Arkansas ban on elementary student access to vending machines during school hours can go further and restrict access to less healthful products for populations in which the information failures are greatest.

First-best policy options for addressing obesity-related financial externalities can alter the relative prices of more and less healthful foods and beverages in a way that can result in more healthful diets and improved weight outcomes. For example, taxes on sugar-sweetened beverages or energy-dense, low-nutrient foods can increase the prices of these products, reduce their consumption, and generate revenues that can subsidize the consumption of fruits, vegetables, whole grains, and other more healthful options (11). Populations that are most likely to have time inconsistent preferences (eg, the young, people who are less educated, people who are socioeconomically disadvantaged) are also more responsive to changes in prices, taxes, and subsidies; therefore, such fiscal interventions can also be effective in addressing this market failure (6,11). Likewise, fiscal policies can be used to address the externalities arising from lack of access to more healthful foods and beverages; tax breaks and other subsidies can be provided to supermarkets that will not otherwise locate in food deserts.

## Conclusion

Although such policies can be effective in correcting for market failures, they raise their own ethical concerns, as Phillips et al (2) describe for the policies contained in Arkansas Act 1220. The ethical concerns are likely to be even more relevant for broader population-based policies than they are for school-based and other policies that target children. Taxes on sugar-sweetened beverages will affect all who drink these beverages, not just people who are obese or at risk for obesity and whose health care is paid for by publicly funded programs. Restrictions on advertising raise concerns about First Amendment rights to commercial free speech. Menu-labeling laws that apply only to larger chain restaurants create an uneven playing field and leave independent restaurants the option of continuing not to provide the information that consumers need to make more informed choices.

The forum on childhood obesity and ethics, sponsored by the Robert Wood Johnson Foundation, that produced this set of articles generated discussions about effective public policies for reducing obesity and its consequences. Similar discussions will be essential to ensure that public policies balance the protection of public health and the rights of individuals.
